# PD1^+^CD8^+^ Cells Are an Independent Prognostic Marker in Patients with Head and Neck Cancer

**DOI:** 10.3390/biomedicines10112794

**Published:** 2022-11-03

**Authors:** Barbora Pokrývková, Marek Grega, Jan Klozar, Ondřej Vencálek, Jaroslav Nunvář, Ruth Tachezy

**Affiliations:** 1 Department of Genetics and Microbiology, Faculty of Science, Charles University, BIOCEV, 252 50 Vestec, Czech Republic; 2 Department of Pathology and Molecular Medicine, 2nd Faculty of Medicine, Charles University, 150 06 Prague, Czech Republic; 3 Department of Otorhinolaryngology and Head and Neck Surgery, 1st Faculty of Medicine, Charles University, University Hospital Motol, 150 06 Prague, Czech Republic; 4 Department of Mathematical Analysis and Applications of Mathematics, Faculty of Science, Palacky University in Olomouc, 771 46 Olomouc, Czech Republic

**Keywords:** head and neck cancer, PD-1, survival, HPV

## Abstract

Head and neck squamous cell carcinomas (HNSCCs) belong to a group of diverse tumors, which can be induced by infection with human papillomavirus (HPV) or tobacco and alcohol consumption. The viral etiology of HNSCC relates to better clinical outcomes reflecting a different immune system response. Here, we retrospectively analyzed 97 tissue samples from oral and oropharyngeal carcinomas associated and non-associated with HPV infection using multispectral fluorescent immunohistochemistry. To evaluate the immune cell infiltration in tumor and stroma compartments, we designed four panels of four to five antibodies. We detected more T lymphocytes in the stroma, compared to the tumor parenchyma. In HPV positive (HPV^+^) in comparison to HPV negative (HPV^−^) tumors, higher counts of CD3^+^CD4^+^, CD3^+^CD8^+^, PD1^+^CD4^+^, PD1^+^CD8^+^ T cells, and ICOS^−^ Treg cells were detected while more ICOS^+^ Treg cells and CTLA4^+^CD4^+^ T cells were observed in HPV^−^ than in HPV^+^ tumors. The results of the univariate and multivariate analyses confirmed the predominant impact of HPV status on prognosis. More importantly, the number of CD8^+^PD-1^+^ T cells was identified as an independent factor, influencing the overall and/or disease-specific survival of patients with oral cavity or oropharyngeal carcinomas.

## 1. Introduction

Head and neck squamous cell carcinomas (HNSCCs) are the world’s sixth most common cancer with locations in the oropharynx, hypopharynx, nasopharynx, larynx, oral cavity, and nasal cavity [1]. Historically, most HNSCCs were associated with tobacco and alcohol consumption; however, in recent decades, a growing number of oropharyngeal (OP) and oral cavity (OC) tumors are caused by a persistent infection with high-risk human papillomavirus (HPV) [1,2]. Patients with HPV-positive (HPV^+^) tumors are younger and, compared to patients with non-virally induced tumors (HPV^−^), have better prognosis, reflecting their improved treatment response [3,4,5].

One of the factors that can explain the difference in clinical outcomes between patients with different tumor etiology is their immune response. The phenotypes and numbers of tumor-infiltrating lymphocytes (TIL) as one of the components of the tumor microenvironment (TME) were described as a significant prognostic factor in many tumors, including HNSCC [5,6,7,8,9]. Moreover, the level of CD3^+^ and CD8^+^ T lymphocytes in a tumor invasive margin in colorectal carcinoma was recently introduced as an immunoscore, which serves as a prognostic factor for colorectal carcinoma patients and is now approved as an addition to the conventional tumor-node-metastasis (TNM) staging [6]. Multispectral fluorescence immunohistochemistry (fIHC) is a powerful tool for a detailed analysis of the TME. This method allows us to assess the phenotype and quantity of cell types in different compartments of the tumor, since, in comparison to flow cytometry, the architecture of the tissue remains preserved. The fIHC is uniquely suited for the study of the interaction between immune and cancer cells in situ.

CD4^+^ and CD8^+^ T lymphocytes are important TIL subpopulations in the TME. As CD4^+^ T lymphocytes include diverse functional subpopulations, their effect on prognosis is difficult to decipher. Many studies do not analyze T lymphocyte spatial location in the TME and HPV status of HNSCC tumors, which modify the observed correlations [10]. CD8^+^ T cells represent the main anti-tumor TIL population and are considered as a positive prognostic factor in the majority of tumors [10,11,12]. High CD8^+^ TILs infiltration was also shown to be associated with better survival of patients with OP, hypopharyngeal, and laryngeal SCC [9,13]. The anti-tumor function of T cells in TME may be suppressed by the FOXP3^+^ regulatory T cells (Treg) activity or by the PD-1 (programmed cell death 1)/PD-L1 (PD-1 ligand) interactions [14]. Moreover, the expression of the inducible co-stimulator (ICOS) receptor on T cells, including Treg, can regulate the checkpoint inhibitors efficacy [15]. In this retrospective study with a 10-year follow-up, we analyzed the HNSCC TME with respect to the presence of an active HPV infection and prognostic significance of the studied TILs.

## 2. Materials and Methods

### 2.1. Sample Collection, Processing, and Characterization

Samples of primary HNSCC (ICD-10: C01–C06, C09, C10) and non-malignant tonsillar tissue were provided by the Department of Otorhinolaryngology and Head and Neck Surgery, Motol University Hospital, Prague. They were collected, processed, and characterized in previous studies between 2001–2012 [4,16,17]. Briefly, they were screened for the presence and type of HPV and active viral infection and were classified by a pathologist using the TNM nomenclature as valid at the time of patients’ enrollment (7th edition) [18]. For this study, 97 formalin-fixed and paraffin-embedded (FFPE) samples were selected. According to the presence of active viral infection, they were divided into HPV^+^ or HPV^−^. All patients signed an informed consent form and completed a questionnaire about risk factors for HPV infection and HNSCC induction when enrolled in the study approved by the Ethical Committee of the Motol University Hospital in 2001.

### 2.2. Tissue Sections Preparation and Antibodies Validation

From each FFPE tumor tissue block, 2 μm sections were prepared on Superfrost^®^ Plus microscope slides (VWR, Leuven, Belgium) and confirmed by a pathologist for the presence of tumor tissue. The slides were deparaffinized at 60 °C for two hours, washed in xylene for 3 × 10 min, hydrated with descending grades of ethanol (100%, 96%, and 70%; Penta, Prague, Czech Republic), and fixed for 20 min in neutral buffered formalin, pH 7.2–7.4 (Diapath, Martinengo, Italy). Heat-induced epitope retrieval (HIER) was carried out by using microwave (96–98 °C) in buffer of pH 9.0 (AR9, Tris-EDTA; Zytomed Systems, Berlin, Germany) or pH 6.0 (AR6, citrate buffer; Akoya Biosciences, Menlo Park, CA, USA). After 10 min of blocking using Antibody Diluent/Block (Akoya Biosciences), the slides were incubated with the primary antibodies listed in Table 1 and subsequently stained with the Opal™ 7-Color Manual IHC Kit (Akoya Biosciences), according to the manufacturers’ instructions. Validation of the antibodies was carried out on samples of non-malignant tonsillar and HNSCC tissue. To verify staining specificity, the corresponding isotype controls, as well as no-primary controls, were performed using the appropriate isotype antibody or Antibody Diluent/Block, respectively.

### 2.3. Multispectral fIHC Panel Design and Optimization

We designed four different antibody panels. A staining order for primary antibodies was set with respect to different HIER length requirements and stability of the epitopes after multiple HIER treatments. For matching fluorophores with the primary antibodies, possible colocalization of antibodies in the panel was considered. After each round of staining, the complex of primary and secondary antibodies was removed by HIER using the antigen retrieval (AR) buffer, optimal for the following antibody in the panel. To ensure complete removal of the antibodies complex, stripping quality controls were performed. The final settings for each panel with optimized dilutions of antibodies are specified in Table S1. 

### 2.4. Image Acquiring and Processing

All slides were snapped by using the MantraSnap 1.0.3 software included in the Mantra Quantitative Pathology Workstation (Akoya Biosciences) for DAPI, FITC, Cy3, Texas Red, and Cy5 data acquisition. From each tissue section, five different regions of interest were randomly selected with a 20X objective. Images were analyzed using the InForm 2.4.6. software (Akoya Biosciences). Four separated algorithms for trainable tissue and cell segmentation and cell phenotyping were prepared. The tissue was segmented into the tumor parenchyma, stroma, and background compartments and checked by the pathologist. The algorithms were trained to specifically phenotype the cells according to different expression of markers. The optimization workflow of algorithms is listed in Supplement Figure S1. For determining the phenotype of the cells, a phenotyping confidence cut off of 80% was set. The numbers of positive cells of different phenotypes were counted separately per megapixel (Mpx) for the tumor parenchymal and stromal compartments. We introduced several TIL phenotypes for each panel (Table 2). 

### 2.5. Statistical Analysis

The numbers of cells of distinct phenotypes were evaluated separately for the parenchyma and the stroma and counted per Mpx for the five regions of interest. Differences between parenchyma and stroma infiltration were evaluated using the sign test, and the Mann–Whitney U test was used to detect differences between the HPV^+^ and HPV^−^ groups. Patients were grouped based on HPV E6 mRNA detection as HPV^+^ or HPV^−^. 

The Cox proportional hazards model was applied for the overall survival (OS) and disease-specific survival (DSS) analyses. The following factors were included: age, gender, education (≤12 years, >12 years), smoking history (nonsmoker, ex-smoker, smoker), alcohol use (nondrinker, ex-drinker, drinker), tumor location (oropharyngeal, oral), pathological tumor extension (pT1–4), pathological nodal status (pN0–3), tumor stage (S I, II, III, IV), metastasis (pM1), tumor grade (1–3), and relapse (yes, no). As the samples were collected between 2001 and 2012, the tumors were classified according to the TNM 7 staging system, hence, the p16 positive tumors do not have a separate category. Furthermore, the p16 status (p16^+^, p16^−^), HPV status (HPV^+^, HPV^−^), and cell numbers of distinct phenotypes per Mpx separately in the parenchyma and the stroma or in total were evaluated. The zero values in the cell numbers were substituted by the 0.1 value as the lowest non-zero value. The models were analyzed using the Bayesian information criterion (BIC). For all the statistical tests, a *p* value of <0.05 was considered as a significant difference. The statistical analyses were performed using the GraphPad Prism 8.4.2. software (GraphPad Software, San Diego, CA, USA) and R version 4.0.2 (https://www.R-project.org/ accessed on 02 November 2022) [19].

## 3. Results

### 3.1. Patients’ Characterization

From the previously characterized cohort [4,16], 97 patients were chosen according to the HPV E6 expression status and tumor location. The selected group consisted of 78 men (78/97, 80%) and 19 women (19/97, 20%) with a median age of 58 years. The median of follow-up time was 45 months. The patients were divided based on HPV E6 mRNA detection into the HPV^+^ (45/97, 46%) and HPV^−^ (52/97, 54%) groups. Of the 97 patients, 18 relapsed and 43 died. The patient demographics and clinical characteristics are listed in Table 3.

### 3.2. HPV^+^ Tumors Are More Infiltrated by T lymphocytes

**Figure 1:**
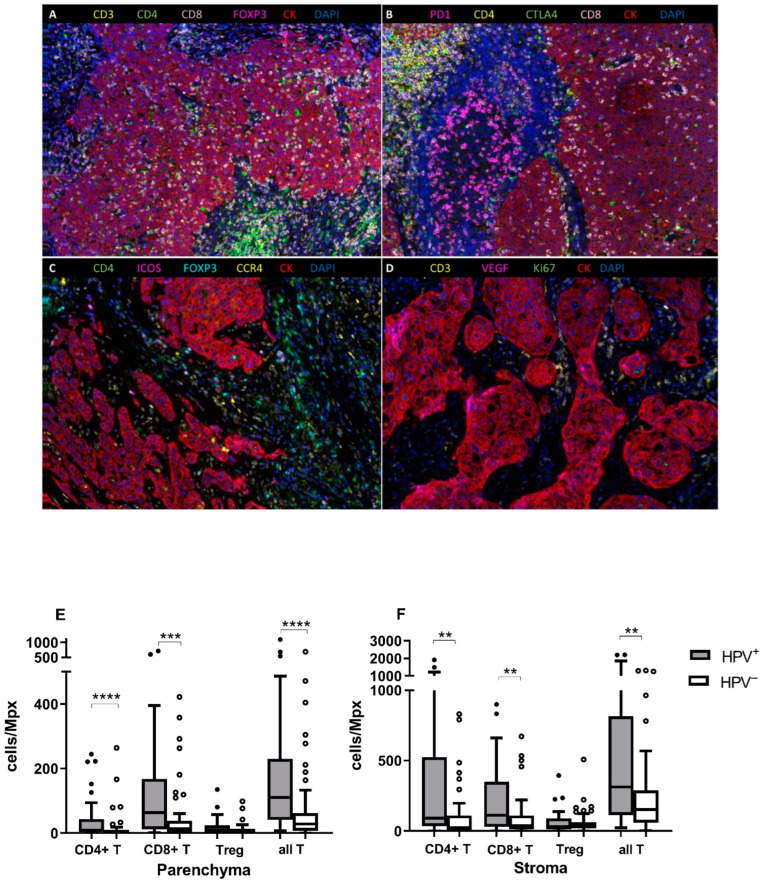
Representative multispectral fIHC staining of FFPE tissue samples with four antibody panels, 20X magnification. (**A**) CD3 (yellow), CD4 (green), CD8 (pink), FOXP3 (magenta), CK (red), and DAPI (blue) staining. (**B**) PD-1 (magenta), CD4 (yellow), CTLA4 (green), CD8 (pink), CK (red), and DAPI (blue) staining. (**C**) CD4 (green), ICOS (magenta), FOXP3 (cyan), CCR4 (yellow), CK (red), and DAPI (blue) staining. (**D**) CD3 (yellow), VEGF (magenta), Ki67 (green), CK (red), and DAPI (blue) staining. T lymphocytes infiltration in the parenchyma and the stroma compartments according to tumor etiology. (**E**) The numbers of CD3^+^CD4^+^, CD3^+^CD8^+^, all CD3^+^ T lymphocytes, and Treg cells per Mpx were compared between the HPV^+^ and HPV^−^ groups in the parenchyma and (**F**) the stroma compartments. Significantly higher infiltration of CD3^+^CD4^+^, CD3^+^CD8^+^, and all CD3^+^ T lymphocytes was observed in the HPV^+^ groups in both compartments. The observed difference for Treg cells was not statistically significant. ** *p* ≤ 0.01, *** *p* ≤ 0.001, **** *p* ≤ 0.0001, Mann–Whitney U test.

We analyzed 97 FFPE samples with four antibody panels using multispectral fIHC (Figure 1A–D). For panel 2, three samples were excluded due to poor quality of staining. Regardless of the tumor status, the higher infiltration with CD3^+^CD4^+^ (Th), CD3^+^CD8^+^ (Tc), and Treg cells was detected in the stroma, compared to the parenchyma (*p* < 0.0001 for all cell populations, data not shown). For all measured populations, we observed a highly or moderately positive correlation of TIL counts/Mpx between the parenchyma and the stroma compartments (Table S2). Next, we analyzed the data with respect to tumor etiology. Significantly higher infiltration by all T cells (sum of Th, Tc, and Treg cells), Th cells, and Tc cells was detected both in the parenchyma and the stroma of HPV^+^ tumors (all T cells *p* < 0.0001 for parenchyma, *p* = 0.0014 for stroma; Th *p* < 0.0001 for parenchyma, *p* = 0.0017 for stroma; Tc *p* = 0.0003 for parenchyma, *p* = 0.0093 for stroma). The Treg counts did not statistically differ between the two tumor compartments neither in the HPV^+^ nor in HPV^−^ groups. However, we observed statistically non-significantly higher Treg counts in HPV^+^ patients (Figure 1E,F).

### 3.3. PD-1^+^CD4^+^ and PD-1^+^CD8^+^ Levels Are Higher in HPV^+^ Tumors

**Figure 2:**
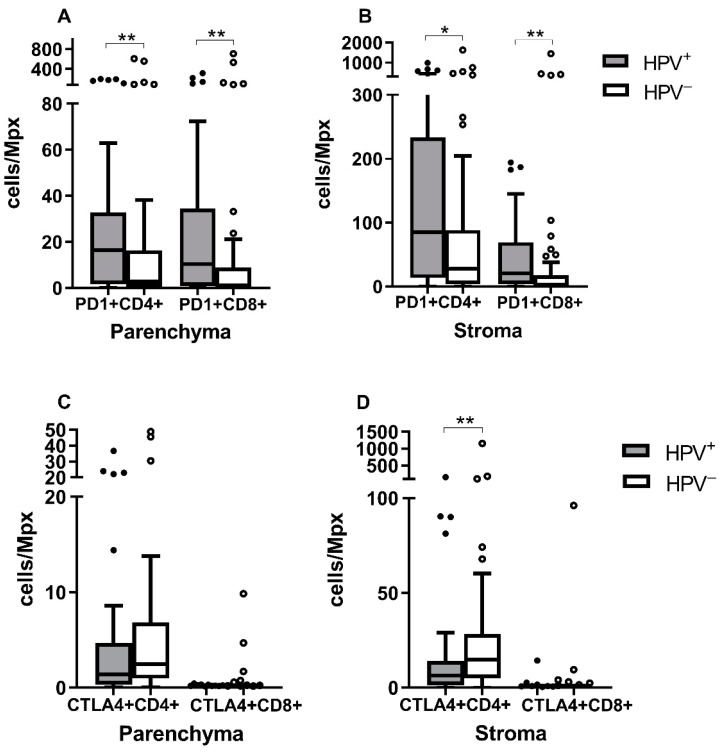
PD-1^+^ or CTLA4^+^ T lymphocyte infiltration in different compartments of the tumors of different etiology. The numbers of CD4^+^ and CD8^+^ T lymphocytes expressing the PD-1 molecule in (**A**) the parenchyma and (**B**) the stroma, and the numbers of CD4^+^ and CD8^+^ T lymphocytes producing the CTLA4 molecule in (**C**) the parenchyma and (**D**) the stroma were compared between groups. PD-1 expression was significantly higher in both compartments of HPV^+^ tumors, while the CTLA4 production was significantly higher only in the stroma of HPV^−^ tumors. * *p* ≤ 0.05, ** *p* ≤ 0.01, Mann–Whitney U test.

To determine the functional status of CD4^+^ and CD8^+^ TILs, we detected the expression of PD-1 and CTLA4 proteins. As shown in Figure 2A,B, significantly higher quantities of PD-1^+^CD4^+^ and PD-1^+^CD8^+^ T cells were observed both in the parenchyma and the stroma of HPV^+^ tumors than in HPV^−^ tumors (PD-1^+^CD4^+^ *p* = 0.0092 for parenchyma, *p* = 0.0125 for stroma; PD-1^+^CD8^+^ *p* = 0.0039 for parenchyma, *p* = 0.0055 for stroma). Increased levels of CTLA4^+^CD4^+^ T cells were detected in the stroma and parenchyma of HPV^−^ tumors, but the difference was statistically significant only for the stroma (*p* = 0.0021). No difference was observed in the CTLA4^+^CD8^+^ T cell numbers between the two groups in either the parenchyma or the stroma (Figure 2C,D).

### 3.4. ICOS^+^ Treg Are More Abundant in HPV^−^ Tumors

**Figure 3:**
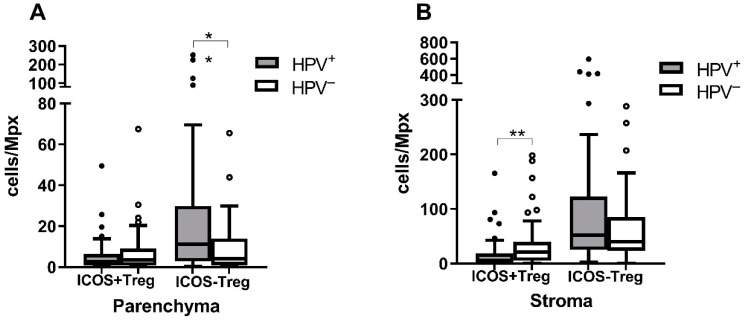
Comparison of the ICOS^+^ and ICOS^−^ Treg levels in the HPV^+^ and HPV^−^ tumors. The numbers of Treg cells with or without ICOS production per Mpx were compared in (**A**) the parenchyma and (**B**) the stroma of the tumors of different etiology. Higher ICOS^+^ Treg levels were observed in both the parenchyma and stroma of the HPV^−^ tumors, with the difference being significant for the stroma. Higher ICOS^−^ Treg numbers were detected again in both the parenchyma and stroma of HPV^+^ tumors, with the difference being significant for the parenchyma. * *p* ≤ 0.05, ** *p* ≤ 0.01, Mann–Whitney U test.

As we did not observe differences in Treg numbers between the HPV^+^ and HPV^−^ groups, we compared the levels of the Treg subpopulation, producing the costimulatory molecule ICOS (ICOS^+^FOXP3^+^CD4^+^). The ICOS^+^ Treg subpopulation was more abundant in the HPV^−^ than in the HPV^+^ tumors (*p* = 0.0034 for the stroma, *p* = 0.4334 for the parenchyma) while the ICOS^−^ Treg (ICOS^−^FOXP3^+^CD4^+^) numbers were higher in the HPV^+^ tumors (*p* = 0.2158 for the stroma, *p* = 0.0044 for the parenchyma) (Figure 3).

### 3.5. The Infiltration Level of T Cell Subpopulations Is Prognostic for OS and DSS

To evaluate the influence of TIL infiltration on prognosis, we used Cox models. We fitted 38 models for OS and DSS analyses. Models with and without HPV status and with the evaluation of the parenchyma and stroma separately or combined were tested. The models with the best BIC score are listed in Table 4, and the remaining models are shown in Table S3. In the models with HPV involvement and parenchyma/stroma distinction for OS (BIC = 308.96), the HPV RNA positivity appeared to be the strongest predictive factor for OS, and the lower tumor stage and higher number of parenchymal PD-1^+^CD8^+^ T cells related to better OS (Table 4). The best model for DSS (BIC = 169.16) includes a lower CD4^+^/Treg ratio, which was associated with better survival, along with the positive HPV RNA status and a higher number of stromal PD-1^+^CD8^+^ T cells (Table 4).

In the models where the IHC markers were evaluated without tissue segmentation but HPV status was included, HPV RNA positivity, higher number of all PD-1^+^CD8^+^ T cells, and lower tumor stage or nodal status were connected with better OS and DSS, and low number of all CTLA4^+^CD8^+^ T cells was linked to better OS (Table 4 and Supplement Table S3).

In models not including the HPV status, the main predictors of worse OS were positive smoking history, a lower number of parenchymal PD-1^+^CD8^+^ T cells, a higher number of all CTLA4^+^CD8^+^ T, and tumor size or tumor stage. Similarly, for DSS, a lower number of stromal PD-1^+^CD8^+^ T cells, together with the number of stromal CD8^+^ T cells, and higher age related to worse survival (Table 4 and Table S3).

We have shown the level of stromal or parenchymal PD-1^+^CD8^+^ T cells to be a positive prognostic predictor in all models. Additionally, rising tumor stage or nodal status appeared to be a negative predictor for both OS and DSS (Table S3). Our data suggest that the level of PD-1^+^CD8^+^ TIL can serve as an independent prognostic marker for HNSCC patients and may improve the current prognosis assessment.

## 4. Discussion

The major aim of this retrospective study was to analyze the tumor microenvironment in HNSCC of different etiology in order to explain the better prognosis of patients with HPV-positive tumors and to evaluate non-viral markers in the populations of TILs as a possible independent prognostic tool for HNSCC patients. 

The prognostic significance of the TIL count was evaluated in several malignancies and recently this parameter has been approved as an additional characteristic to the TNM classification for colorectal carcinomas [5,6,7,8,9]. It has also been proposed that the spatial distribution of TILs in the tumor parenchyma or stroma relates to their different biological function in the process of tumorigenesis [20]. 

In our study, HPV-induced tumors were more infiltrated by CD3^+^CD4^+^ and CD3^+^CD8^+^ T cells. Higher infiltration by T cells of HPV^+^ tumors than HPV^−^ tumors may be explained by the presence of viral antigens, which stimulate the immune response more strongly. Despite the differences in their designs, several studies have come to similar results. Badoual et al. found higher CD4^+^ T cell counts in the stroma of HNSCC tumors [21]. Other studies regardless of the method used detected a statistically significantly higher infiltration of CD3^+^, CD4^+^, and CD8^+^ T lymphocytes in both the parenchyma and the stroma of HPV^+^ tumors [22,23,24,25,26,27,28]. Although we observed higher Treg cell infiltration in HPV^+^ tumors, the difference from HPV^−^ tumors was not statistically significant. The same was shown by IHC [25], gene expression [28], and flow cytometry analyses [27,29]. Significantly higher Treg counts in the HPV^+^ cohort, in comparison to HPV^−^ tumors, were only reported by a single study for tonsillar SCC [23]. To analyze Treg cells more thoroughly, their functional status was evaluated according to the expression of the ICOS molecule. ICOS serves as a co-stimulatory receptor of immunogenic T cells, but it is also involved in the activation of Treg cells resulting in the immunosuppression [15]. ICOS^+^ Treg cells were more prevalent in the stroma and parenchyma of HPV^−^ tumors, but the difference was statistically significant only for the stroma. Inversely, ICOS^−^ Treg counts were higher in both compartments of HPV^+^ tumors with a statistically significant difference for parenchyma location. To our knowledge, this is the first study analyzing the spatial prevalence of ICOS on Treg cells in HNSCC of different etiology, and the results suggest that these cells can have different function in HPV^+^ and HPV^−^ tumors. 

Furthermore, we have detected significantly higher counts of PD-1^+^CD4^+^ and PD-1^+^CD8^+^ T cells in both the parenchyma and the stroma of HPV^+^ tumors, compared to HPV^−^ tumors. Higher levels of PD-1^+^CD4^+^ and/or PD-1^+^CD8^+^ T cells and PD-1 mRNA were also observed in HPV^+^ than in HPV^−^ HNSCC by others [27,30,31]. It was also shown that PD-1 expression was more frequent on CD4^+^ than CD8^+^ T cells in HNSCC [21,32], which agrees with our study. Oguejiofor et al. observed a higher level of PD-1^+^CD8^+^ cells in the stroma, compared to the parenchyma but with no difference in the PD-1^+^CD8^+^ counts between HPV^+^ and HPV^−^ OPSCC [22]. 

Thanks to the long-term follow-up, we were able to explore the impact of TILs infiltration on prognosis, which is the main benefit of this study. We fitted several models, including clinicopathological characteristics, HPV RNA status, and tissue compartment. We were also interested if any of the studied population of TILs would be an independent prognostic marker for HNSCC regardless of the HPV association. The influence of TILs on prognosis was also analyzed by others who, unfortunately, did not take account of HPV status and/or spatial distribution was not considered, which makes the comparison of results difficult. We found that a higher number of parenchymal or stromal PD-1^+^CD8^+^ T cells is associated with both better OS and DSS of OC and OPSCC patients in all of the models. When HPV status was included, the multivariate analysis confirmed HPV positivity as the strongest independent positive prognostic factor for OS and DSS [3,4]. 

In OC and OPSCC, a higher number of CD8^+^ T cells was associated with better OS or DFS [23,24,33,34,35]. However, other studies did not confirm this observation [30,36,37,38]. The interplay between subfamilies of TILs in different tumor compartments is as complex as the outcome of these interactions. Therefore, it is important to analyze TILs more deeply with respect to their functional status. The PD-1 molecule is expressed by activated T lymphocytes, pointing to the persistent antigen stimulation, and subsequent T cell exhaustion. However, in the TME context, the PD-1 expression may not be followed by the expression of co-inhibitory molecules and, therefore, is not a marker of T cell exhaustion [30,39]. In the HPV^−^ cohort, it has been suggested that PD1^+^CD8^+^ TIL cells act as a tumor-specific population, compared to the PD1^−^CD8^+^ TILs and play a key anti-tumor role [39]. It was also shown that high infiltration by PD-1^+^CD4^+^ T cells in HPV^+^ tumors and by PD-1^+^CD8^+^ T cells in HPV^+^ and HPV^−^ tumors related to better OS than low infiltration by these cell populations [30,39]. The increased numbers of PD-1^+^CD8^+^ T cells in tumors suggest that patients contain tumor-specific T cells that can be activated and—before silencing by inhibitory molecules, such as PD-1—exert an antitumor effect, supplementing both radiotherapy and surgery and enhancing survival. Due to the limited number of markers, which can be detected using multispectral fIHC, we are not able to characterize the PD-1^+^CD8^+^ T cells in the more detailed manner, which is the limitation of this study. To describe the status of PD-1^+^CD8^+^ T cells in the TME, expression of other activation and/or exhaustion markers can be examined on a fresh tumor tissue, using other methods, such as flow cytometry. However, the results of our study and of others indicate that this marker should be included in the prognostic immunoscore for HNSCC [30,39]. The multivariate analyses did not reveal the influence of the counts of CD4^+^ T lymphocytes on prognosis, which was also observed by others [24,30]. In our study, the levels of PD-1, CTLA4, and ICOS-expressing CD4^+^ T cells were analyzed in the context of prognosis, and none of these subpopulations influenced OS or DSS. 

The level of Treg cells itself did not affect OS or DSS but was positively correlated with a better locoregional control in HNSCC [21]. It was proposed that ICOS^+^ Treg cells in the TME have higher immunosuppressive capacity than the ICOS^−^ Treg population [15]. The higher ICOS^+^ Treg counts found in the HPV^−^ tumors may relate to worse outcomes of HPV^−^ patients. In our study, despite the findings of higher ICOS^+^Treg cell counts in HPV^−^ tumors, no influence of this subpopulation on OS or DSS was detected. Similar to Lukesova et al. who observed the phenomenon in the blood, in our models, we found that a low CD8^+^/FOXP3^+^ ratio related to improved OS and DSS [40]. In the study of de Ruiter et al., the CD8^+^/FOXP3^+^ ratio measured in HPV^−^ tumor tissue microarray cores did not corelate with improved survival [41]. However, in agreement with our study, Nasman identified a high CD8^+^/FOXP3^+^ ratio, connected with better DFS in both HPV^+^ and HPV^−^ groups [23]. 

## 5. Conclusions

In this study, we evaluated the level of TILs in different compartments of the HNSCC microenvironment using multispectral IHC. We compared the TILs levels with respect to the presence of an active HPV infection and tumor compartment in a retrospective cohort of patients under long-term follow-up. The impact of different TIL infiltration levels on prognosis was studied. We revealed that higher levels of PD-1^+^CD8^+^ T lymphocytes improved the prognosis of the patients in all our models, which points out the importance of this marker and entitles it to be included in the prognostic immunoscore for patients with OC and OPSCC of different etiology.

## Figures and Tables

**Table 1:** List of antibodies.

Name	Clone	Manufacturer
CD3e	SP7	ThermoFisher Scientific (Waltham, MA, USA)
CD4	EP204	Zeta Corporation (Arcadia, CA, USA)
CD8-α	C8/144B	Santa Cruz Biotechnology (Dallas, TX, USA)
FoxP3	206D	BioLegend (San Diego, CA, USA)
PD-1	EPR4877(2)	Abcam (Cambridge, United Kingdom)
CTLA4	F-8	Santa Cruz Biotechnology (Dallas, TX, USA)
ICOS	SP98	Abcam (Cambridge, United Kingdom)
VEGF	EP1176Y	Biocare Medical (Pacheco, CA, USA)
Ki67	sc-23900	Santa Cruz Biotechnology (Dallas, TX, USA)
CCR4	polyclonal	Novus Biological (Centennial, CO, USA)
Cytokeratin Pan Type I/II	AE1/AE3	ThermoFisher Scientific (Waltham, MA, USA)

**Table 2:** Immune cell phenotypes in Panels 1–4.

	Markers		Markers
Panel 1	Th: CD3^+^CD4^+^	Panel 3	CD3^+^
	Tc: CD3^+^CD8^+^		Ki67^+^
	Treg: FOXP3^+^CD3^+^CD4^+^		VEGF^+^
Panel 2	CD4^+^	Panel 4	CD4^+^
	PD-1^+^CD4^+^		FOXP3^+^CD4^+^
	CTLA4^+^CD4^+^		ICOS^+^CD4^+^
	CD8^+^		ICOS^+^FOXP3^+^CD4^+^
	PD-1^+^CD8^+^		CCR4^+^
	CTLA4^+^CD8^+^		CD4^+^FOXP3^+^CCR4^+^

Abbreviations: Th—helper T, Tc—cytotoxic T, Treg—regulatory T, PD-1—programmed cell death 1, CTLA4—cytotoxic T lymphocyte antigen 4, VEGF—vascular endothelial growth factor, ICOS—inducible T cell co-stimulator, CCR4—C-C chemokine receptor 4.

**Table 3:** Patient demographics and clinical characteristics.

Characteristics		Total	HPV^+^	HPV^−^
		No. (%)	No. (%)	No. (%)
No. of patients		97 (100%)	45 (46%)	52 (54%)
Age	Mean age	57.23	57.98	56.58
(years)	Median age	58	59	56
Gender	female	19 (20%)	10 (22%)	9 (17%)
	male	78 (80%)	35 (78%)	43 (83%)
Tumor location	oropharynx	83 (86%)	45 (100%)	38 (73%)
	oral cavity	14 (14%)	0 (0%)	14 (27%)
Education ^a^	>12 years	33 (34%)	18 (41%)	15 (29%)
	≤12 years	63 (66%)	26 (59%)	37 (71%)
Smoking	never	19 (20%)	15 (33%)	4 (8%)
	past	32 (33%)	19 (42%)	13 (25%)
	current	46 (47%)	11 (25%)	35 (67%)
Alcohol	never	20 (21%)	12 (27%)	8 (15%)
consumption	past	13 (13%)	7 (16%)	6 (12%)
	current	64 (66%)	26 (57%)	38 (73%)
No. of sex partners	>7	41 (46%)	15 (35%)	26 (55%)
	≤6	49 (54%)	28 (65%)	21 (45%)
Tumor size	T1	17 (18%)	7 (16%)	10 (19%)
(pT)	T2	61 (63%)	27 (60%)	34 (65%)
	T3	13 (13%)	9 (20%)	4 (8%)
	T4	6 (6%)	2 (4%)	4 (8%)
Nodal status	N0	31 (32%)	9 (20%)	22 (42%)
(pN)	N1	16 (16.5%)	5 (11%)	11 (21%)
	N2	47 (48.5%)	28 (62%)	19 (37%)
	N3	3 (3%)	3 (7%)	0 (0%)
Metastasis	0	97 (100%)	45 (100%)	52 (100%)
(M)	1	0 (0%)	0 (0%)	0 (0%)
Tumor stage	I	5 (5%)	1 (2%)	4 (8%)
(S)	II	24 (25%)	6 (13%)	18 (35%)
	III	16 (16%)	7 (16%)	9 (17%)
	IV	52 (54%)	31 (69%)	21 (40%)
Tumor grade	1	14 (14%)	3 (7%)	11 (21%)
	2	57 (59%)	25 (55%)	32 (62%)
	3	26 (27%)	17 (38%)	9 (17%)
Relapse	no	82 (85%)	41 (91%)	41 (79%)
	yes	15 (15%)	4 (9%)	11 (21%)

^a^ For one patient, data were not available.

**Table 4:** Hazard ratio (HR) values for cell populations from the best models influencing overall survival (OS) and disease-specific survival (DSS).

Models Including HPV Status, IHC Markers Evaluated with Respect to the Stromal or Parenchymal Location.			
OS		DSS	
BIC = 308.96	HR (*p* value)	BIC = 169.16	HR (*p* value)
HPV RNA^+^	0.26 (0.0003)	HPV RNA^+^	0.15 (0.0011)
Increasing tumor stage	1.55 (0.0179)	CD3^+^CD4^+^/Treg ratio	3.18 (0.0016)
Parenchymal PD1^+^CD8^+^ T	0.53 (0.0003)	Stromal PD1^+^CD8^+^ T	0.44 (0.0007)
**Models including HPV status, the IHC markers counted regardless of location.**			
OS		DSS	
BIC = 307.31	HR (*p* value)	BIC = 171.50	HR (*p* value)
HPV RNA^+^	0.26 (0.0004)	HPV RNA^+^	0.22 (0.0057)
Increasing tumor stage	1.57 (0.0160)	CD3^+^ T	2.79 (0.0094)
PD1^+^CD8^+^ T	0.52 (0.0001)	PD1^+^CD8^+^ T	0.36 (0.0001)
**Models without HPV status inclusion, the IHC markers evaluated with respect to the stromal or parenchymal location.**			
OS		DSS	
BIC = 313.13	HR (*p* value)	BIC = 174.57	HR (*p* value)
Smoking (No)	0.37 (0.0055)	Increasing age	0.93 (0.0032)
Increasing tumor size	1.44 (0.0709)	Stromal CD8^+^ T	2.19 (0.0165)
Parenchymal PD1^+^CD8^+^ T	0.52 (0.0003)	Stromal PD1^+^CD8^+^ T	0.30 (0.0000)

## Data Availability

The data are publicly available on Zenodo. Barbora Pokryvkova, Marek Grega, Jan Klozar, Ondrej Vencalek, Jaroslav Nunvar, Ruth Tachezy (2022), “PD1+CD8+ cells are an independent prognostic marker in patients with head and neck cancer”, doi: 10.5281/zenodo.6874821.

## Supplementary Materials

The following supporting information can be downloaded at: https://www.mdpi.com/article/10.3390/biomedicines10112794/s1, Table S1:Reaction conditions for each panel of antibodies; Table S2: Cell counts/Mpx in the parenchyma and stroma of HNSCC tumors; Table S3: Models of DSS and OS; Figure S1: Optimization of algorithms for automatic picture analysis.
