# Supplementary material for: PD1+CD8+ Cells Are an Independent Prognostic Marker in Patients with Head and Neck Cancer
Source: Biomedicines. 2022 Nov 3;10(11):2794. doi: 10.3390/biomedicines10112794 (PMC9687997; doi:10.3390/biomedicines10112794)
Supplement: Supplementary file 1 [file biomedicines-10-02794-s001.zip › Figure S1.pdf]

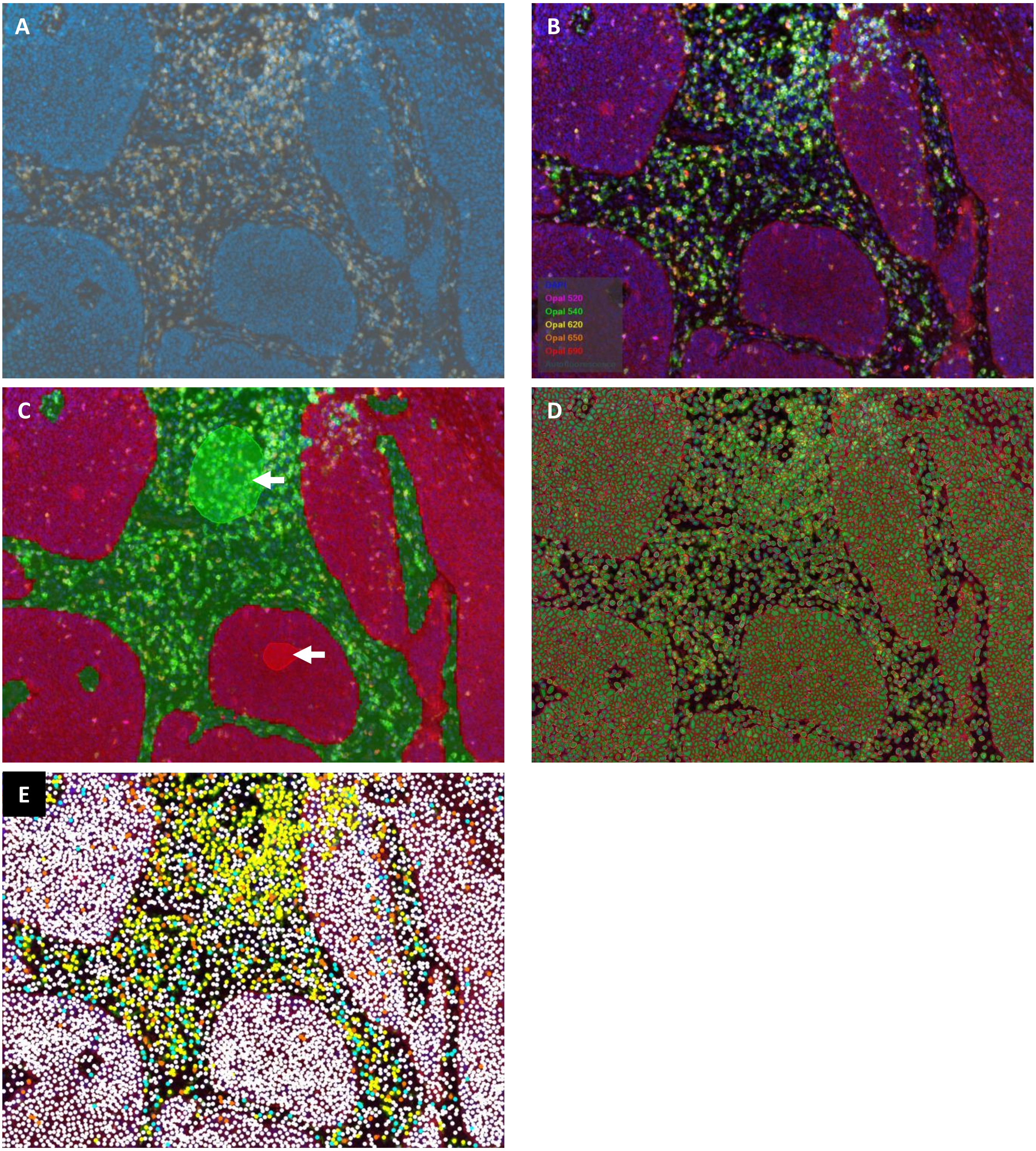

Figure S1. Optimization of algorithms for automatic picture analysis. (**A**) Unprocessed raw image snapped by MantraSnap software with 20x magnification in DAPI, FITC, Cy3, Texas Red, Cy5 acquiring filters. (**B**) Spectrally unmixed image obtained by InForm 2.4.6. software using prepared spectral libraries. The spectral libraries were prepared in InForm 2.4.6. software according to the manufacturers' instructions. (**C**) Tissue segmentation to tumor parenchyma (red), stroma (green). Training regions (white arrows) were selected according to the pan cytokeratin (CK) antibody staining, the components for training were DAPI and the Opal690 signal (CK antibody). The training accuracy was measured to 99,9%, the segmentation resolution was set to „medium" with the minimum segment size to 400 px. (**D**) Cell segmentation to nucleus, cytoplasm, and membrane. The nuclei were recognized by the DAPI signal (relative DAPI intensity 0.21, the splitting sensitivity 0.12, minimal nucleus size 35 px). The cytoplasm was recognized by cytoplasmatic CK antibody staining and the membrane by CD4 antibody staining. The cytoplasm thickness was set to 2 px, and the membrane search distance to 3 px. (**E**) The phenotyping of the cells. The cells for software training were selected manually according to the expression of the markers (in this case: yellow – CD3+CD4+ cells, orange – CD3+CD8+ cells, cyan – CD3+CD4+FOXP3+ cells, white – remaining „other" cells). Several rounds of training were performed, and re-training was done by correcting the cells' phenotype to ensure the high confidence of phenotyping.
