# Supplementary material for: PD1+CD8+ Cells Are an Independent Prognostic Marker in Patients with Head and Neck Cancer
Source: Biomedicines. 2022 Nov 3;10(11):2794. doi: 10.3390/biomedicines10112794 (PMC9687997; doi:10.3390/biomedicines10112794)
Supplement: Supplementary file 1 [file biomedicines-10-02794-s001.zip › Tables S1 and S2.pdf]

Table S1. Reaction conditions for each panel of antibodies. The order of antibodies and buffer used for epitope retrieval (AR) are indicated as well as the reaction conditions for the primary antibody and OPAL fluorophore staining. For all slides, Opal Polymer HRP Ms+Rb as the secondary antibody and DAPI as the final nuclei counterstain were used.

**Panel 1**							**Panel 2**						
#	*AR*		*Antibody*		*OPAL*		*#*	*AR*		*Antibody*		*OPAL*	
		Marker	*Diln.*	*Incubation*	*λ[nm]*	*Diln.*			*Marker*	*Diln.*	*Incubation*	*λ[nm]*	*Diln.*
1	9	CD3e	1:500	1 h/RT	620	1:100	1	9	PD-1	1:50	OVN/4°C	520	1:50
2	9	CD4	1:200	OVN/4°C	540	1:100	2	9	CD4	1:200	OVN/4°C	620	1:100
3	9	CD8-α	1:500	1 h/RT	650	1:100	3	9	CTLA4	1:100	OVN/4°C	540	1:100
4	6	FoxP3	1:50	1 h/RT	520	1:100	4	9	CD8-α	1:500	1 h/RT	650	1:100
5	6	CK	1:800	1 h/RT	690	1:200	5	6	CK	1:800	1 h/RT	690	1:200
**Panel 3**							**Panel 4**						
#	*AR*		*Antibody*		*OPAL*		*#*	*AR*		*Antibody*		*OPAL*	
		Marker	*Diln.*	*Incubation*	*λ[nm]*	*Diln.*			*Marker*	*Diln.*	*Incubation*	*λ[nm]*	*Diln.*
1	9	CD3e	1:400	1 h/RT	620	1:100	1	9	CD4	1:200	OVN/4°C	540	1:100
2	9	VEGF	1:50	OVN/4°C	520	1:100	2	9	ICOS	1:50	OVN/4°C	520	1:50
3	6	Ki67	1:100	1 h/RT	540	1:100	3	6	FoxP3	1:50	1 h/RT	570	1:100
4	6	CK	1:800	1 h/RT	690	1:200	4	6	CCR4	1:200	1 h/RT	620	1:100
							5	6	CK	1:800	1 h/RT	690	1:200

Abbreviations: # - serial No. of antibodies, AR – antigen retrieval buffer pH, Diln. – dilution, RT – room temperature, OVN – overnight

Table S2. Cell counts/Mpx in the parenchyma and stroma of HNSCC tumors.

Panel	*Cell population*	**Parenchyma**				**Stroma**				*Parenchyma-Stroma correlation (r)*
		Zeros (%)*	*Min. nonzero value*	*Median*	*Max.*	*Zeros* (%)*	*Min. nonzero value*	*Median*	*Max.*	
1	CD3+CD4+ (Th)	6	0.27	5.01	264.2	1	0.46	53.85	1912.8	0.71
	CD3+CD8+ (Tc)	3	0.17	29.42	714.56	1	0.28	52.42	899.09	0.83
	CD3+CD4+FOXP3+(Treg)	9	0.21	8.24	135.58	2	0.69	35.6	507.13	0.68
	Th + Tc + Treg sum	0	0.17	53.42	1094.88	0	2.77	205.95	2205.32	0.76
2	CD4+	2	0.17	7.95	213.54	0	1.63	134.67	1563.11	0.70
	PD-1+CD4+	7	0.22	4.62	607.33	9	0.53	37.76	1630.56	0.80
	CTLA4+CD4+	11	0.17	2.09	48.85	6	0.45	9.23	1148.64	0.73
	CD8+	14	0.18	4.94	318.1	5	0.54	41.91	907.32	0.81
	PD-1+CD8+	23	0.2	2.55	709.31	21	0.25	6.41	1445	0.85
	CTLA4+CD8+	79	0.16	0	9.85	76	0.24	0	96.14	0.49
3	CD3+	2	0.48	35.46	465.77	0	0.46	192.92	1628.73	0.64
	Ki67+	27	0.18	0.82	684.08	47	0.21	0.3	656.79	0.69
	VEGF+	14	0.19	5.9	446.56	29	0.29	1.25	904.2	0.44
4	CD4+	6	0.2	4.47	599.06	0	0.33	117.77	2055.96	0.71
	FOXP3+CD4+	7	0.19	5.98	252.41	1	2.11	46.83	596.52	0.77
	ICOS+CD4+	10	0.17	2.99	125.25	8	0.25	10.64	668.6	0.76
	ICOS+FOXP3+CD4+	15	0.17	3.25	67.54	6	0.28	10.16	198.21	0.77
